# Novel Antifungal Agents and Their Activity against *Aspergillus* Species

**DOI:** 10.3390/jof6040213

**Published:** 2020-10-09

**Authors:** Roya Vahedi-Shahandashti, Cornelia Lass-Flörl

**Affiliations:** Institute of Hygiene and Medical Microbiology, Medical University Innsbruck, 6020 Innsbruck, Austria; Roya.Vahedi@i-med.ac.at

**Keywords:** novel antifungal drugs, new therapies, invasive fungal infections, aspergillosis

## Abstract

There is a need for new antifungal agents, mainly due to increased incidence of invasive fungal infections (IFI), high frequency of associated morbidity and mortality and limitations of the current antifungal agents (e.g., toxicity, drug–drug interactions, and resistance). The clinically available antifungals for IFI are restricted to four main classes: polyenes, flucytosine, triazoles, and echinocandins. Several antifungals are hampered by multiple resistance mechanisms being present in fungi. Consequently, novel antifungal agents with new targets and modified chemical structures are required to combat fungal infections. This review will describe novel antifungals, with a focus on the *Aspergillus* species.

## 1. Introduction

Invasive fungal infections (IFI) are responsible for over one million deaths annually, which is a significant global health problem [1,2]. This is mainly due to the increasing number of immunocompromised individuals with altered immune function including primary immune deficiency, cancer chemotherapy, HIV/AIDS, hematologic and solid organ transplantation, prematurity, and immune-modulatory medications [3,4,5]. The number of at-risk patients and improved diagnostic techniques resulted in an alarming rise in the number of reported fungal infections [6,7]. Invasive aspergillosis, an infection caused by fungi of the *Aspergillus* taxon, remains a significant threat, particularly in immunosuppressed patients [8]. The most prevalent *Aspergillus* species are *A. fumigatus*, *A. flavus*, *A. terreus*, and *A. niger* [9]. *Aspergillus* spp. have the capacity to cause a broad range of clinical diseases, from mild and superficial infections, to life-threatening and invasive illnesses with more than 80% mortality rate [10,11]. Pulmonary aspergillosis is considered the most prevalent manifestation of invasive aspergillosis [12].

Antifungal drug discovery has been stagnant in the past. Hence, therapeutic possibilities for IFI are limited [13]. There are currently four major classes of antifungal agents used in the treatment of systemic mycoses: polyenes, azoles, echinocandins and flucytosine [14]. These antifungals have several limitations such as toxicity, drug–drug interactions, variable pharmacokinetics, and reduced bioavailability. The emergence of drug resistance has introduced further limitations [15]. Voriconazole is recommended for first-line treatment followed by liposomal amphotericin B and isavuconazole [16,17]. The rate of azole-resistant *Aspergillus* isolates has increased noticeably, contributing to therapeutic failures [18]. The prevalence of azole-resistant clinical isolates of *Aspergillus* spp. has reached 30% in some regions in Europe, with data outside Europe varying between 0.6% and 11.2% [19].

In this review, we evaluate new antifungal compounds and natural products with a focus on *Aspergillus* spp. Additionally, potential new pathways will be discussed as promising targets.

## 2. Improving Existing Antifungals

This part will briefly address new formulations of existing antifungals, which are active against *Aspergillus* spp.

**Figure 1:**
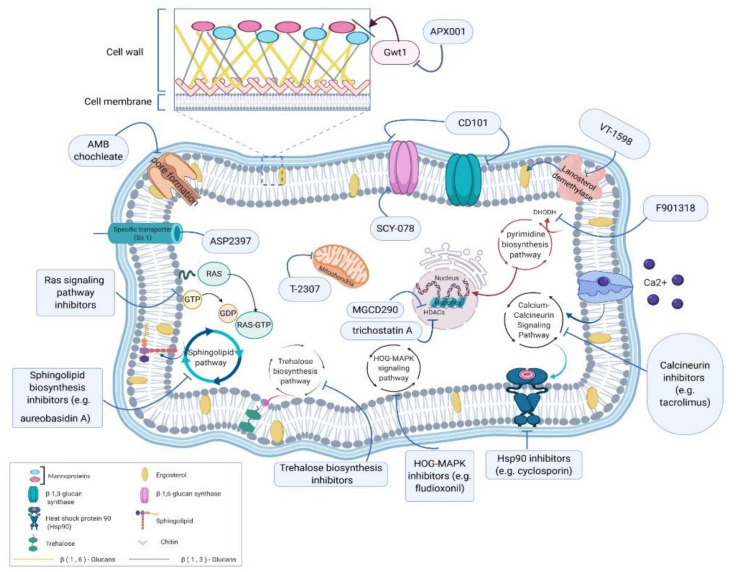
Target sites and potential pathways of the novel antifungals. This diagram of a fungal cell indicates various molecules that can be tackled by antifungal agents (blue boxes), including cell wall, cell membrane, and also intracellular targets such as mitochondria and processes like metabolisms and stress responses.

A summary of agents is given along with their mechanism of action, in vitro activity, and clinical trial phase in Table 1. An overview of different fungal targets is given in Figure 1.

### 2.1. CD101

CD101 (Cidara Therapeutics) (Biafungin) (Rezafungin), a novel structurally formulated echinocandin, for both intravenous and oral use, is a cyclic hexapeptide with a choline moiety at the C5 ornithine position [54]. This drug is presently in phase III human clinical trials.

Echinocandins act as inhibitors of the 1, 3-β-d-glucan synthase enzyme complex, which play a role in the synthesis of fungal cell walls [55]. Compared with other echinocandins, the advantage of CD101 over existing echinocandin drugs lies in its pharmacokinetics. The distinct structural feature of CD101 confers improved stability, resulting in an extended half-life and an enhanced safety profile [54,55,56]. Relative to the 24 h half-life of anidulafungin, CD101 has a half-life of approximately 130h in humans [57]. CD101 is being developed as a weekly intravenous drug for the treatment and prevention of IFI, replacing the daily doses currently available [54].

Compared to other echinocandins, CD101 has shown an enhanced in vitro potency with minimum effective concentrations 90 (MEC90) of ≤0.008–0.03 μg/mL against *Aspergillus* spp., wild-type and azole non-wild-type isolates, including *A. fumigatus* (minimum inhibitory concentration (MIC)50/90, ≤0.008/0.015 μg/mL), *A. terreus* (MIC50/90, 0.015/0.015 μg/mL), *A. niger* (MIC50/90, ≤0.008/0.03 μg/mL) and *A. flavus* (MIC50/90, ≤0.008/≤0.008 μg/mL) [58].

Rezafungin showed in vivo efficacy in a neutropenic murine model with disseminated infection caused by *A. fumigatus* (ATCC 13073), providing 10-day survival rates with a one-time dose of 2 mg/kg [66].

### 2.2. SCY-078 (Formerly MK-3118)

SCY-078 (Scynexis), a class of semisynthetic derivative of the natural product enfumafungin, is a first-in-class orally formulated inhibitor of active β-1,3-glucan synthase, with in vitro and in vivo activity against *Aspergillus* spp. [59,60]. This drug is now in phase III of clinical trials. β-1,3-d-glucan synthase is a unique membrane-associated protein complex in fungi that require the synthesis of the main constituent of the cell wall, β-1,3-d-glucan polymers.

Echinocandins also target the fungal cell wall by blocking β-1,3-d-glucan synthase, but SCY-078 is structurally distinct from the currently available echinocandins, constricting its effectiveness to echinocandin-resistant *Aspergillus* spp. [61,67,68,69].

In vitro activity of SCY-078 demonstrates a promising potency against the *Aspergillus* spp. complex, with the MEC90/MIC90 value (μg/mL) levels remaining below 0.5 µg/mL, for *A. fumigatus* (0.25 µg/mL), *A. terreus* (0.12 µg/mL), *A. flavus* (0.12 µg/mL) and *A. niger* (0.06 µg/mL) [60,61].

The efficacy of SCY-078 has further been shown in a neutropenic mouse model of invasive aspergillosis caused by wild type and azole-resistant *A. fumigatus* strains, leading to an increased survival rate at 15 mg/kg/day and 20 mg/kg/day [70].

### 2.3. Amphotericin B (AMB) Renovated Structure

AMB-deoxycholate has a potent wide-spectrum fungicidal activity, which prompted the development of safer and more effective derivatives. The mode of action of AMB remains unclear, but it is generally believed that it acts via two major mechanisms: an increase in permeation by binding to the ergosterol of fungal membrane, forming trans-membrane channels leading to leakage of cell constituents and a pro-oxidant effect causing oxidative damage in target fungal cells [35,36]. The affinity of AMB to ergosterol-containing membranes is stronger than cholesterol-containing membranes. However, it also has the ability to bind to cholesterol, leading to toxicity in mammalian cells.

To develop a soluble and less-toxic formula, AMB has been structurally modified and conjugated with various soluble macromolecules such as nanoparticle suspensions and conjugated polysaccharides (AMB-arabinogalactan or AMB-polyethylene glycol), leading to lower toxicity and high efficacy in vivo [40,41,42,43,44]. Alternative structures might have different strategies for improving the cellular selectivity or accessing certain body compartments to reduce membrane toxicity of AMB [37]. One proposed mechanism is through minimizing the disruptive power of aggregated forms by attaching to an umbrella-derived molecule and increasing the selectivity of AMB to target fungi [37].

Unlike the existing formulations of AMB, which are only approved for intravenous injection, an oral drug delivery consisting of AMB cochleate lipid–crystal nanoparticles (Matinas BioPharma) is a remarkable improvement as it is structurally more stable against degradation by the gastrointestinal tract [38,39]. AMB cochleate is made up of phosphatidylserine with phospholipid-calcium precipitates, constructing a multilayered structure with a solid, lipid bilayer, with no inner aqueous space [39].

In vivo, orally administered AMB-cochleate has shown high efficacy in a murine model of systemic aspergillosis resulting in 70% survival rates (20 and 40 mg/kg/day of body weight/day for 14 days) [71]. This drug is now in phase II of clinical trials (NCT02971007 and NCT02629419).

## 3. New Antifungal Compounds with Novel Targets in *Aspergillus*

The focus of this section describes targets and mechanisms of action of new anti-*Aspergillus* compounds that have reached the early stages of human clinical testing.

### 3.1. T2307

T-2307 or 4-{3-[1-(3-{4-[amino(imino)methyl] phenoxy} propyl) piperidin-4-yl] propoxy} benzamidine is a novel arylamidine structure that belongs to the category of aromatic diamidines and is synthesized by the Toyama Chemical Company [20]. In 2015, phase I safety trials in healthy young and elderly volunteers were examined. (clinicaltrials.gov Identifier: NCT02289599).

The specific structure of the amidine (functional group) of T-2307 is associated with its distinct mechanism of action [72]. In *Candida albicans*, this component is selectively transported in through a high-affinity spermine and spermidine transportation system (polyamine transporters) regulated by *Agp2* [21]. The high affinity of transporters in *C. albicans* for T-2307 compared to other diamidines such as pentamidine, leads to different rates of uptake, which may lead to the different in vitro antifungal activities [22].

Furthermore, the selectivity of the mechanism of action of T-2307 in fungal and mammalian cells may reflect the high affinity with fungal mitochondria. Once it accumulates inside the fungal cells, T-2307 results in the collapse of the mitochondrial membrane respiration potential, eventually leading to fungicidal activity [73].

T-2307 exhibits broad-spectrum activity against most of the clinically relevant pathogenic yeasts and filamentous fungi including *Aspergillus* spp., *Candida* spp., and *Cryptococcus neoformans*, with notably low MICs [20]. The MIC of T-2307 against *Aspergillus* spp. ranged from 0.0156 to 1 μg/mL, which is similar to those of voriconazole and micafungin. However, T-2307 indicates fungicidal activity against *A. nidulans* and *A. niger* (0.0313 to 0.0625 μg/mL) whereas it is categorized as fungistatic against other *Aspergillus* spp. (4 to >64 μg/mL).

In vivo activity in a murine model with systemic infection caused by *A. fumigatus* resulted in more than 80% survival rate (1 mg/kg). Nevertheless, T-2307 in a disseminated candidiasis mouse model was more effective than micafungin and amphotericin B but in a disseminated aspergillosis mouse model was comparable to the activities of micafungin and amphotericin B [20].

### 3.2. Fosmanogepix (APX001)

APX001 (formerly E1210, 2-amino-3-(3-{4-[(pyridine-2-yloxy) methylbenzyl}-1-2-isoxazol-5-yl) pyridinium-1-yl] methyl hydrogen phosphate) is a small cell wall-active antifungal compound discovered by Eisai Company (Tokyo, Japan), which is being synthesized by Amplyx Pharmaceuticals, SanDiego, CA. APX001, an N-phosphonooxymethyl, is a prodrug that is rapidly broken down by systemic alkaline phosphatases to the active component, APX001A [23,74].

Phase 1 clinical trials have shown safety in both healthy volunteers and patients with severe leukemia [74]. Phase II studies are ongoing.

E1210/APX001 demonstrates selective antifungal activity by inhibiting fungal adhesion and invasion. Glycosylphosphatidylinositol (GPI) cell wall anchor proteins, known as mannoproteins, display various functions ranging from enzymatic activity, signaling, cell adhesion, cell wall metabolism, and immune response [75]. In *C. albicans*, GPIs mediate cross-linking of cell wall mannoproteins to β-1,6-glucan, preserve the integrity of the fungal cell wall and play a role in adhesion. APX001 targets the fungal enzyme GPI-anchored wall transferase (Gwt1), subsequently inactivating an early step of posttranslational modification of GPI anchor proteins [24,76,77]. Since Gwt1 catalyzes the inositol acylation of fungus-specific GPI, Gwt1 inhibition leads to the disruption of GPI-anchored protein maturation. A lack of these proteins in *C. albicans* revealed that the cell wall weakens, resulting in β-1, 3-glucan exposure, hyphal growth suppression and increase in the recognition of the fungus by immune cells [25]. Interestingly, even though mammals possess the Gwp1 homolog gene, PigW, E1210 is only active against fungal Gwt1ps. E1210 has no inhibitory effect on human inositol acylation, rendering it an effective therapeutic target for fungal infections [25].

APX001A is active against a broad range of pathogenic yeast and molds, including *Aspergillus* spp., *Fusarium* spp., and black molds [24]. The in vitro activity of E1210 and comparator antifungal agents (e.g., caspofungin, itraconazole, posaconazole and voriconazole) against wild-type, polyene- and triazole-resistant strains of *Aspergillus* spp., including *A. flavus*, *A. fumigatus*, *A. niger*, *A. terreus* exhibited an exceptional potency in vitro (i.e., MIC≤0.06 μg/mL) [24]. Compared to reference antifungals (e.g., voriconazole, caspofungin, liposomal amphotericin B), E1210 showed higher efficacy and improved survival rate in murine pulmonary aspergillosis models [78]. Animal models (rats and monkeys) indicate that APX001 was rapidly absorbed and widely distributed for oral and intravenous administration [78].

### 3.3. ASP2397 (VL-2397)

ASP2397 is a cyclic hexapeptide natural antifungal compound, cyclo{Asn-Leu-dPhe-[(N5-acetyl-N5-hydroxyOrn)3]-} Al(III), derived from *Acremonium persicinum* [26].

In a phase I study, healthy volunteers who received single or multiple intravascular doses of increasing concentrations displayed adequate tolerance of up to 1200 mg without any accumulation [79]. Phase II clinical trials (registration no. NCT03327727) focused on the treatment of patients with acute leukemia suffering from invasive aspergillosis and bone marrow transplantation. However, this study was prematurely terminated due to a financial decision [80,81].

ASP2397 has the ferrichrome type siderophore structure, a low-molecular-weight siderophore with high specificity for iron [82]. Siderophores (iron chelators) are produced by microorganisms in response to iron deficiency. Under iron-deficient conditions, they increase their uptake of iron by enhancing the expression of the siderophore transporter [83,84]. The exact mechanism of ASP2397 within fungal cells remains unknown. Nevertheless, ASP2397 is believed to localize within fungal cells, such as *A. fumigatus*, via the uptake of siderophore iron transporter 1 (Sit1) [27]. It is shown that the presence of an iron chelator in medium intensifies the potent antifungal activity of ASP2397 against *Aspergillus* spp. Hence, the study proposes the uptake of ASP2397 by specific siderophore iron transporter 1 (Sit1) in *A. fumigatus* as a potential antifungal therapeutic approach. Inactivation of Sit1 results in resistance to ASP2397 [85]. Complementary investigations revealed that an additional intracellular target of ASP2397 probably exists. This may be explained by the observation that AS2524371, an ASP2397 analog with a similar siderphore structure, with the exception of an amino acid substitution (Gly-Ser-Gly replaces Asn-Leu-dPhe), has no antifungal activity against *A. fumigatus* [26]. The in vitro testing of ASP2397 showed excellent fungicidal efficacy against most *Aspergillus* spp., including wild-type strains as well as azole-resistant mutants of *A*. *fumigatus*, *A. terreus*, *A. flavus* and *A. nidulans.* With the exception of *A. niger*, the MIC ranged between 1 to 4 μg/mL in human serum [27,85].

In contrast to liposomal amphotericin B and azoles (voriconazole and posaconazole), ASP2397 has a faster and more effective fungicidal activity in vitro. This fungicidal activity has also been observed against germinated conidia of some *Aspergillus* spp. in vitro in human serum, suggesting that it may produce more suitable therapeutic outcomes for patients with invasive aspergillosis [27].

Remarkably, ASP2397 showed excellent in vitro efficacy against wild-type strains as well as azole-resistant mutants of *A. fumigatus* (Cyp51A). However, MICs of both ASP2397 and azoles were elevated in the isolate of *A. terreus* with M217I CYP51A mutation [28]. Compared to *A. fumigatus*, higher MIC of ASP2397 in *A. terreus* isolates may suggest that it is less effective against this species. Since CYP51A is not the target of ASP2397, further studies are required to investigate the underlying mechanisms behind the MIC elevation against mutant *A. terreus* isolate.

The effect of delayed treatment of ASP2397 in an in vivo mouse model of invasive aspergillosis showed a high survival rate (100% survival), compared to posaconazole (40% survival rate) [27]. Given that ASP2397 has no target in mammalian cells, and possesses distinct modes of antifungal mechanisms compared to azoles and amphotericin B, it is hypothesized that ASP2397 will have selective fungal toxicity and could be a promising substitute for the treatment of azole-resistant *Aspergillus* infections.

### 3.4. F901318 (F2G) or Olorofim

F901318, a representative member of a novel class of antifungal drugs, the orotomides, clinically exhibits exceptional potency against a broad range of dimorphic and filamentous fungi, particularly *Aspergillus spp*. [29]. It is currently in phase III of clinical trials and in phase II as an oral and intravenous agent with a specific emphasis on aspergillosis (NCT0286178).

The novel mechanism of action of F901318 is well described in *A. fumigatus* [29]. It targets the dihydroorotate dehydrogenase (DHODH) enzyme, which in *Aspergillus* spp. is encoded by the pyre gene. DHODH is an oxidoreductase enzyme that catalyzes the fourth step of the de novo pyrimidine biosynthesis pathway, the reduction of dihydroorotate to orotate. Pyrimidines are crucial subunits for the synthesis of DNA and RNA and for the formation of precursors for lipid and carbohydrate metabolism. Despite the presence of a mammalian version of the enzyme, F901318 has differential inhibitory activity. It is 2000-fold more potent against fungal DHODH than the mammalian enzyme homolog. Supplementary protein kinetic experiments demonstrated that F901318 is a reversible inhibitor of *A. fumigatus* DHODH, which competitively inhibits the ubiquinone (coenzyme Q) cofactor [29].

This compound with such a unique target displayed potent in vitro and in vivo activity against several medically relevant molds, including several *Aspergillus* spp. However, it is not effective against *Mucorales*, *C. neoformans* and *Candida* spp. [29,30,31,32]. The lack of activity against *Candida* spp., *C. neoformans*, and *Mucorales* is due to a phylogenetically distant DHODH, although olorofim susceptible organisms are classified together under the same DHODH phylogenetic tree [29].

F901318 demonstrates efficacy against *Aspergillus* spp., regardless of species and methods used, which is of significant importance given the increased prevalence of azole-resistant *Aspergillus* spp. [86,87]. MIC ranges of F901318 (0.002–0.063 μg/mL) against *Aspergillus* spp. including *A. fumigatus* (azol-resistant and non-azole resistant strains), *A. terreus*, *A. flavous*, *A. nidulans*, *A. tubingensis* and *A. tubingensis* are relatively low compared to MICs of various azoles and amphotericin B [30,31]. Resistance induction to olorofim assessed by serial passage and drug gradients has no influence, at least on *A. fumigatus* [29].

The efficacy of olorofim therapy (15 mg/kg, three times per day) against infection with *A. fumigatus*, *A. nidulans* and *A. tanneri* in both neutropenic and chronic granulomatous disease (CGD) mouse models exhibited promising therapeutic outcomes. A 10-day survival rate of 80% to 88% and 63% to 88% in the neutropenic mouse model and CGD mouse model was reported, respectively [87].

The narrow-spectrum activity of F901318 forces an additional study design with the use of another antifungal until the specific diagnosis of aspergillosis is reached.

### 3.5. VT-1598

VT-1598, an investigational tetrazole from Viamet Pharmaceuticals, is a selective fungal cytochrome P51 (CYP51) enzyme inhibitor with clinically significant reduced drug–drug interactions. This compound is currently in phase I of clinical trials [33]. Two main limitations of azole class antifungals reinforced this developing new formulation: non-selective activity and drug–drug interaction of the members of this class. Azoles generally inhibit fungal 14α-lanosterol demethylase (aka Cyp51), a key cytochrome P450 enzyme (CYP450) in ergosterol biosynthesis, resulting in a deficiency of ergosterol and accumulation of toxic 14α-methylated sterols in membranes [88]. Furthermore, azoles can hinder other cytochrome P450 enzymes, conducting non-specific activity of this class of antifungals. The clinical concentrations of some azoles can be influenced by drugs that inhibit or induce the activity of CYP 450 enzymes since some azoles are substrates of these enzymes.

In *A. fumigatus*, VT-1598 is a structurally distinct CYP51 inhibitor in that its triazole metal-binding group is substituted with tetrazole, resulting in more specific inhibition of fungal Cyp 51 enzymes [89]. The X-ray molecular structure of a VT-1598/*A. fumigatus* CYP51 complex has explained that the improved hydrogen bond between the phenoxymethyl oxygen of VT-1598 and the imidazole ring nitrogen of His374 of CYP51 residue is associated with maximal efficacy and broad-spectrum activity of VT-1598 [90].

VT-1598 demonstrated a comparable in vitro activity against *Aspergillus* spp. with similar geometric mean (GM) MICs to those posaconazole and voriconazole for *A. flavus* (0.685 μg/mL), *A. niger* (1.78 μg/mL) and *A. terreus* (0.533 μg/mL) [34]. In contrast, wild-type isolates of *A. fumigatus* showed higher GM MICs ranges of VT-1598 (0.25–2 μg/mL) in comparison to those of posaconazole and voriconazole. However, the GM MICs of VT-1598 against *A. fumigatus* CYP51A mutants with elevated posaconazole and voriconazole MICs showed a noticeably reduction (13.3 μg/mL).

An in vivo 12-day survival study in a disseminated mouse model of invasive aspergillosis showed 100% survival at 20 and 40 mg/kg with a suppressed fungal burden [91].

## 4. Potential Pathways as Targets against *Aspergillus*

The following studies discuss fungal molecules displaying potential targets for developing new antifungal agents with limited or no damage to host cellular functions.

### 4.1. Calcium–Calcineurin Signaling Network

Calcium signal transduction in fungi has gained importance due to its crucial role in the survival and adaptation of fungi. Calcium, a second messenger molecule, plays direct roles in fungal physiological processes, mediates stress responses, and promotes virulence [92,93,94,95,96]. Calcineurin, one of the regulators of calcium homeostasis with a subtle structural difference in fungi compared to humans, is a potential target of selective inhibitors that could potentially be used in antifungal therapy [45].

Calcineurin, a conserved Ca^2+^-calmodulin (CaM) activated protein phosphatase 2B, belongs to the phospho-protein phosphatase family. The stress response in the fungal cell, including *Saccharomyces cerevisiae* and *Schizosaccharomyces pombe* is initiated by Ca^2+^ uptake, which then binds to the binding sites of calmodulin. After a conformational transition, Ca^2+^-bound calmodulin forms a ternary complex together with the calcineurin subunits, CnA and CnB. The calmodulin–CnA–CnB complex acquires a phosphatase activity and dephosphorylates the transcription factor Crz1. Genes activated by dephosphorylized Crz1 are involved in calcium-dependent signaling and regulation of several essential cellular processes in many pathogenic fungi including growth, septation, morphological states transition, cell wall integrity, virulence, stress responses, and drug resistance [46,97]. Furthermore, calcineurin is associated with heat shock protein 90 (Hsp90) and histone deacetylases (HDACs, also referred to as lysine deacetylases, KDACs). The molecular chaperone Hsp90 activates its target protein, calcineurin phosphatase, which plays a key role in stress responses and cell wall repair mechanisms induced by antifungals exposure [98,99,100]. The mechanisms displayed by calcineurin instigate the emergence and maintenance of drug resistance in various fungal species [101,102]. HDACs play an essential role in fungal virulence by controlling the expression and function of multiple proteins, including chaperones, such as Hsp90, and secondary metabolites that are important for basal growth or stress adaptation [103].

Hsp90 has been identified to have a principal role in the acquisition and evolution of resistance to azoles and echinocandins. Importantly, growing evidence suggests that this inherent resistance mechanism is mediated via calcineurin [101,104,105,106].

Therefore, targeting the Hsp90–calcineurin axis may be a promising antifungal strategy and enhance the activity of different classes of antifungal drugs, such as the cell-wall-acting echinocandins and the ergosterol biosynthesis inhibitor azoles. Several studies have demonstrated that immunosuppressive drugs exert antifungal effects against a variety of pathogenic fungi by inhibiting calcineurin signaling network and related components. Although these compounds are all currently in clinical use as immunosuppressive therapy and anti-proliferative agents, the potent immunosuppressive activity of these drugs hindered their expansion as antifungal agents. Thus, the following section analyzes some immunosuppressive drugs which have the potential to play a role in antifungal therapy.

#### 4.1.1. Tacrolimus (FK506)

Tacrolimus (FK506 or Fujimycin), a macrolide lactone extracted from Streptomyces tsukubaensis, is commonly used as an immunosuppressive drug in transplantation [107]. The potential mechanism of tacrolimus in *A. fumigatus* is suggested through its binding to the intracellular protein FKBP12, preventing the calcineurin signal pathway, a principal component in the regulation of intracellular Ca^2+^ concentration [46]. The FKBP12–tacrolimus complex suppresses the phosphatase activity of the calmodulin–CnA–CnB complex, resulting in the inhibition of the transcription factor calcineurin-responsive zinc finger 1 (Crz1) and corresponding stress-related genes. Subsequently, calcineurin inhibitors, like tacrolimus, may function as potent antifungals and may reverse resistance against standard antifungal drugs or increase their efficacy [108,109].

Notably, in vitro antifungal efficacy of FK506 towards planktonic cells and biofilm of *Aspergillus* spp. have been investigated, showing variable susceptibility [45,47,48]. Among the *Aspergillus* spp., most of the examined *A. fumigatus* and *A. terreus* isolates displayed MECs of 0.025–0.05 μg/mL. FK506 was especially effective against *A. niger* (0.006–0.012 μg/mL), reaching >90% growth inhibition. However, *A. flavus* isolates tended to have higher MECs (0.1–0.2 μg/mL), and no substantial impact of FK506 was recognized against *A. ustus* or *A. versicolor* [49]. Combinations of tacrolimus with voriconazole or AMB show synergistic inhibitory activity against *Aspergillus* spp. biofilms [47]. Furthermore, there is an in vitro fungicidal synergism between FK506 and the normally fungistatic caspofungin against *A. fumigatus*, resulting in delayed filamentation and the production of even smaller hyphae [110].

In vivo, FK506 showed improved survival rates in an invasive aspergillosis CD-1 mouse model (1 mg/kg of body weight/day), compared to those treated with cyclosporin A. Nevertheless, higher doses of FK506 (10 mg/kg of body weight/day) resulted in a significant decrease in survival rates. It is suggested that higher doses of FK506 trigger an immunosuppressive effect, thereby offsetting the drug’s moderate anti-aspergillosis activity [111].

Calcineurin inhibitors, such as FK506 and cyclosporine A, decrease the immune response by suppressing T cell proliferation such as interferon gamma (IFNγ) [111,112], thereby increasing overall survival rate [111]. Since IFNγ plays a role in invasive aspergillosis [112], administration of FK506 and cyclosporine A may decrease the risk of serious *Aspergillus* infections and provide a better protection in transplant individuals [50]. Patients under immunosuppressant therapy are at a higher risk of acquiring invasive aspergillosis. Therefore, it is essential to determine the optimal dose for the co-administration of immunosuppressants and antifungal drugs in these patients.

#### 4.1.2. Cyclosporin A

Cyclosporins, a family of lipophilic cyclic undecapeptides metabolites, are produced by the filamentous fungi, *Trichoderma polysporum*. Cyclosporin A, the main representative of the Cyclosporin family, is a calcineurin inhibitor with a potent immunosuppressive and antifungal activity, either on its own or in combination with existing antifungals [113,114]. Cyclosporin, in combination with the antifungals caspofungin or itraconazole, showed in vitro synergy against *A. fumigatus* [114].

Since calcineurin inhibitors pose immunosuppressive effects, Hsp90 inhibitors might provide more beneficial strategies.

#### 4.1.3. Geldanamycin

Geldanamycin is a member of the ansamycin antibiotic family with anti-tumor activity [51]. Geldanamycin acts as an Hsp90 inhibitor by preventing the chaperone activity of the Hsp90 by competing for ATP binding [115]. Hsp90 accelerates the development of drug resistance by triggering new mutations that have immediate phenotypic consequences. Abrogation of resistance in fungi by Hsp90 inhibitors has been suggested as a new therapeutic strategy [101]. The Hsp90 inhibitor geldanamycin and its derivatives, 17-(allylamino)-17-demethoxygeldanamycin (17-AAG) and 17-dimethylaminoethylamino-17-demethoxygeldanamycin (17-DMAG), display modest in vitro activity against molds, with an MEC ≥50% range of 0.015–16 μg/mL [49].

Nevertheless, the combination of geldanamycin with caspofungin in *A. fumigatus* results in fungicidal activity, as well as in azole-resistant strains [110]. Moreover, combination therapy of geldanamycin and caspofungin in the *Galleria mellonella* model has enhanced the survival rate of larvae with *A. fumigatus* infections [105]. Prior to this study, *A. fumigatus* has been shown to be lethal in larvae, despite monotherapy with each agent, including azole-resistant strains [105]. It has also been shown that geldanamycin enhances the efficacy of caspofungin against *A. fumigatus* and *A. terreus* by the synergistic effect [116].

#### 4.1.4. Trichostatin A

Hindering HDACs in pathogenic fungi establishes a promising therapeutic strategy. It was showcased in *A. nidulans, A. oryzae,* and *A. fumigatusas* as an epigenetic therapy via the modified expression of related genes of virulence or drug resistance, by regulating chromatin structure and transcription through lysine deacetylation of histones [103]. HDACs eliminate acetyl groups from lysines on core histones and other cellular proteins that are involved in gene regulation of stress responses [103]. HDACs have been associated with virulence, expression and regulation of essential drug resistance-related proteins, such as the chaperone Hsp90 protein and drug efflux pumps [52,53].

Trichostatin A, a broad spectrum HDAC inhibitor, exhibits variable antifungal activity against clinical isolates of *Aspergillus* spp., including azole-resistant *A. ustus* [106]. In vitro, trichostatin A has shown weak activity against *A. fumigatus* and *A. flavus* isolates, but had better activity against 90% of *A. niger*, *A. terreus*, *A. versicolor* and *A. ustus* isolates with an MEC of 2 μg/mL [106]. Concerning antifungal interactions, synergistic activity between trichostatin A and caspofungin was observed against some *Aspergillus* spp. [49,117].

#### 4.1.5. MGCD290

MGCD290 is another selective HDAC inhibitor, which shows a high MIC (8 to >32 μg/mL) against molds, including *Aspergillus* spp. However, it acts synergistically with azoles (voriconazole and posaconazole) against azole-resistant fungal isolates [118].

### 4.2. Ras and Sphingolipid Synthesis Pathways

In *C. neoformans,* it has been shown that two signaling pathways of Ras and sphingolipid synthesis are necessary for the efficient propagation of infections [119,120]. Concerning the role of these pathways in virulence and structural differences of these molecules in mammalian and fungal cells, blocking the synthesis and/or function of these pathways has emerged as possible a unique target for the development of new drugs. Sphingolipids, a major class of eukaryotic lipids, present a variety of roles in fungal cellular metabolisms, heat stress response, signal transduction and virulence [62,121,122].

Various studies in *Candida* and *Aspergillus* spp. have recently shown that plasma membrane sphingolipids, such as inositol phosphoryl ceramide and glucosylceramide, have essential roles in fungal pathogenesis and drug resistance [63,64,123,124]. Targeting the enzymes involved in the sphingolipid biosynthetic pathways via inhibition or gene deletion can potentially reduce the virulence of fungal pathogens, including *Aspergillus* spp. [62,122]. Several new sphingolipid inhibitors have been discovered, which reduce the levels of fungal sphingolipids in contrast with mammalian cells such as Aureobasidin A, d-threo-1-phenyl-2-palmitoyl-3-pyrrolidinopropanol, d-threo-3′,4′-ethylenedioxy-P4), *N*′-(3-bromo-4-hydroxybenzylidene)-2-methyl benzohydrazide and 3-bromo-*N*′-(3-bromo-4-hydroxybenzylidene) benzohydrazide) [65,124,125,126].

The Ras family are a cluster of membrane-associated guanosine triphosphatase (GTPase) proteins that play a major role in signal transduction pathways in eukaryotic cells. Ras proteins are stimulated by binding to guanosine nucleotide exchange factors (GEFs) and are inactivated by interaction with GTPase activator proteins (GAPs) [127]. Mature Ras proteins could be transported to the plasma membrane via two pathways, either through trafficking by the secretory system in their palmitoylated form or being non-palmitoylated by a non-classical pathway [128].

It has been described that a lack of Ras signaling pathways in *A. fumigatus* through palmitoylation-driven inhibition, results in decreased fungal growth, decreased cell wall integrity, and loss of virulence [129,130]. Ras signaling pathways have been investigated extensively for developing anticancer therapeutics. Since the mechanisms of Ras activation and post-translational modifications are common in both humans and fungi, this information could be translated into novel strategies in treating *Aspergillus* infections. Several antifungal targets within the Ras signaling pathway could be of interest, including a) hindering Ras Proteins and their interactions [131], b) inhibition of Ras post-translational modifications such as farnesylation, and [132] c) inhibition of palmitoylation [133].

Therefore, designing or discovering inhibitors with high selectivity to fungal homologs could be a promising therapeutic approach.

### 4.3. Trehalose Synthesis Pathway

Trehalose, a non-reducing disaccharide, acts as a reserve carbohydrate source in cell processes such as glycolysis, sporulation, and germination in fungal spores and vegetative cells, including *A. niger*, *S. cerevisiae*, and *Neurospora crassa* [134]. It functions as a protectant under environmental stress and nutrient limitation [135], maintaining the cell membrane under stressful conditions by interacting with proteins and phospholipids. Therefore, the structure of the membrane under dehydrated conditions and thermal-related stress is preserved [136]. Trehalose biosynthesis is one of the pathways that exist in fungi, including *C. albicans*, *C. neoformans*, and *A. fumigatus* but not in humans, rendering it a promising target for novel antifungal agents [135]. The main characteristics of this pathway are its direct link to glycolysis [137] and the participation of two primary synthesizing enzymes, trehalose-6-phosphate synthase (Tps1), and trehalose-6-phosphate phosphatase (Tps2), which are specific to this path [138].

Several investigations have focused on the trehalose pathway in *Aspergillus* spp. [139,140,141,142], confirming the critical role of this biosynthesis pathway in development, stress response, and pathogenicity. In *A. fumigatus*, the enhanced trehalose content of hyphae is associated with response to heat shock stress, which is correlated with increased expression levels of two putative trehalose-6-phosphate synthase genes, tpsA, and tpsB. It has also been demonstrated that blocking this pathway affects conidial germination, thermo-tolerance, and response to high-level oxidative stress in vitro. Interestingly, mutant strains of tpsA and tpsB resulted in more virulence in a murine model of invasive aspergillosis [141]. Compared to compounds targeting Ras and calcineurin pathways, few inhibitors have been discovered for this pathway, which makes it an attractive potential target of antifungal therapy with few consequences on mammal metabolism and biochemical networks.

### 4.4. High-Osmolarity Glycerol (HOG)-Mitogen-Activated Protein Kinase (MAPK) Signaling Pathway

In *S. cerevisiae*, the HOG-MAPK signaling pathway controls the adaptation to environmental stress and regulation of fungal morphology [143]. The HOG-MAPK pathway consists of two cascades of signaling proteins, the putative membrane protein and the two-component phosphorelay system [144]. Diverse extracellular stimuli activate MAPK cascades through subsequent phosphorylation and MAPK activation, resulting in the activation of transcription factors and the expression of distinct genes [145]. However, the function of the HOG-MAPK pathway in filamentous fungi remains unclear. In *A. fumigatus*, the contribution of the HOG-MAPK pathway has been discovered to influence adaption to thermal stress and susceptibility to itraconazole at high temperature [146].

Further studies showcased that the lack of MAPK in *A. fumigatus* enhanced the sensitivity to oxidative stress induced antifungals, including amphotericin B and itraconazole [147]. The absence of two-component phosphorelay systems of MAPK in humans may provide suitable targets for developing new fungicides without notable toxicity [148]. The HOG pathway has been recognized as the target for fludioxonil, phenylpyrrole fungicide with a broad spectrum, providing insight into novel targets for synergistic antifungal drug combinations [149,150]. It is thought to act by interfering with sugar transport and sugar phosphorylation and by disordering the membrane [151].

The above-mentioned pathways are examples of potential antifungal targets that provide a framework for the development of antifungal compounds.

## 5. Natural Products as Anti-*Aspergillus* Agents

Natural products have been important as a source of bioactive molecules, including potent antifungals [152]. Amphotericin B and caspofungin have been derived from natural sources [153], and several investigations have focused on the screening of natural extracts with antifungal activities [154,155]. Rosemary essential oil (REO) demonstrated in vitro activity against *A. flavous*, with an MIC and minimum fungicidal concentrations (MFC) of 500 µg/mL. Further investigations confirmed changes in fungal morphology and a reduction in ergosterol content, suggesting REO as a potent compound [155]. Moreover, antifungal activity of 82 essential oils against *A. niger*, *C. albicans*, and *C. neoformans* has been investigated [156]. *A. niger* showed weak susceptibility, and 45% of essential oils displayed activity against *C. neoformans* isolates.

Humidimycin, a bacterial ring peptide, has been shown to potentiate the activity of sub-lethal concentrations of caspofungin against *A. fumigatus* isolates [157]. Humidimycin is thought to be involved in HOG pathway inhibition, thereby decreasing the protective stress response induced by caspofungin [157]. Since the HOG pathway has not been found in mammals, humidimycin is a promising compound for further analysis.

## 6. Perspective

Given the rise of antifungal drug resistance, novel antifungal agents and new drug targets are highly needed. Recently, an expanding insight into the fungal cellular structures and related processes has contributed to the development of promising antifungal drugs with a wide therapeutic index and potentially without targeting human cells.

Novel compounds with a broad-spectrum of antifungal activity may overcome the lack of sensitive diagnostic assays and may target drug-resistant representatives. The feasibility and results from ongoing clinical trials are highly awaited.

## Figures and Tables

**Table 1:** Summary of antifungal compounds mentioned in the review.

Class	Antifungal Compound	Mechanism of Action	In Vitro Activity (Minimum inhibitory concentration) (MIC)	Advantage	Clinical Trial Phase	References
Arylamidine	T-2307	Inhibits intracellular mitochondrial membrane respiration potential	0.0156–2 μg/mL *A.fumigatus*, *A. terreus*, *A. flavus*, *A. nidulans* and *A. niger*	◦ Preferential uptake by fungal cells	Phase I	[20,21,22]
Glycosylphosphatidylinositol (GPI) inhibitors	E1210/APX001 (Fosmanogepix)	Inhibition of Gwt1, Glycosylphosphatidylinositol (GPI) anchor protein synthesis	≤0.008-0.25 μg/mL *A.fumigatus, A. terreus, A. flavus* and *A. niger*	◦ Broad spectrum ◦ Fungal-specific target ◦ Synergizes with available antifungal	Phase II planned	[23,24,25]
Siderophore	VL-2397 (ASP2397)	Uptaking by specific siderophore iron transporter (Sit1), but an unknown intracellular target	1-4 μg/mL *A.fumigatus*, *A. terreus*, *A. flavus* and *A. niger*		Phase II	[26,27,28]
Orotomides	F90138 (olorofim)	Inhibition of dihydroorotate dehydrogenase (DHODH) in pyrimidine synthesis	<0.03 µg/mL *A.fumigatus*, *azole-resistant A. fumigatus*, *A. terreus*, *A. flavus* and *A. nidulans*	◦ Oral and intravenous formulation ◦ No reported cross-resistance	Phase III	[29,30,31,32]
Tetrazole	VT-1598	Inhibition lanosterol demethylase	0.25-2 μg/mL *A. fumigatus*	◦ Selectivity for fungal CYP51 ◦ Broad spectrum	Phase I	[33,34]
Polyenes	Amphotericin B (AMB) New formulations	Fungal membrane disruption or Pore formation by binding to ergosterol	0.25–1 μg/mL *A. fumigatus* 1–8 μg/mL *A. fumigatus*	◦ Broad spectrum ◦ Oral administration ◦ Less toxicity	Phase II No human clinical trials	[35,36] [37,38,39] [40,41,42,43,44]
	Coch-AmB AMB-conjugated with polysaccharides					
Calcineurin inhibitors	Tacrolimus (FK506)	Calcineurin Inhibition	0.01–0.6 μg/mL (Minimum effective concentration) (MEC)*A. fumigatus*	◦ Synergizes with caspofungin	No human clinical trials	[45,46,47,48,49,50]
Calcineurin inhibitors	Cyclosporin A	Calcineurin Inhibition	0.5–1 μg/mL (MEC) *A. fumigatus*		No human clinical trials	[50]
Hsp90 inhibitors	Geldanamycin	Heat shock protein 90 (Hsp90) Inhibition	4 μg/mL (MEC) *A. fumigatus*	◦ Synergizes with caspofungin	No human clinical trials	[51]
HDAC inhibitors	Trichostatin A	Histone deacetylase (HDAC) Inhibition	4 μg/mL *A. fumigatus*	◦ Synergizes with caspofungin	No human clinical trials	[52]
HDAC inhibitors	MGCD290	Histone deacetylase (HDAC) Inhibition	8->32 μg/mL *A. fumigatus*	◦ Broad spectrum ◦ Synergizes with approved antifungal	Phase II	[53]
Glucan synthesis inhibitors	CD101 (Biafungin)	1,3-β-d-glucan synthase Inhibition	≤0.008/0.03 μg/mL *A. fumigatus*, *A. terreus*, *A. flavus* and *A. niger*	◦ Improved stability ◦ Long half-life ◦ Safety profile	Phase III	[54,55,56,57,58]
Glucan synthesis inhibitors	SCY-078 (MK-3118)	1,3-β-d-glucan synthase Inhibition	0.03-0.25 µg/mL *A.fumigatus*, *A. terreus*, *A. flavus* and *A. niger*	◦ Oral and IV formulation ◦ Activity against itraconazole-resistant Aspergillus strains	Phase III	[59,60,61]
Glycolipid inhibitors	Aureobasidin A	Inhibition of inositol phosphorylceramide (IPC) synthase, sphingolipid syntheses	4 μg/mL *A. fumigatus*	◦ Synergize with caspofungin	No human clinical trials	[62,63,64,65]

## Abbreviations

IFI	Invasive fungal infection
MICs	Minimum inhibitory concentrations
MEC	Minimum effective concentration
GM	Geometric mean
GPI	Glycosylphosphatidylinositol
DHODH	Dihydroorotate dehydrogenase
HDACs	Histone deacetylases
GAPs	GTPase activator proteins
GEFs	Guanosine nucleotide exchange factors
HOG	High osmolarity glycerol
MAPK	Mitogen activated protein kinase
REO	Rosemary essential oil
MFC	Minimum fungicidal activity
IFNγ	Interferon gamma
Crz1	Calcineurin-responsive Zinc finger 1
Gwt1	GPI-anchored wall transferase 1
GTPase	Guanosine Triphosphatase
